# Diffusion-kurtosis imaging predicts early radiotherapy response in nasopharyngeal carcinoma patients

**DOI:** 10.18632/oncotarget.19820

**Published:** 2017-08-02

**Authors:** Gang Wu, Meng-Meng Li, Feng Chen, Shao-Ming Lin, Kai Yang, Ying-Man Zhao, Xiao-Lei Zhu, Wei-Yuan Huang, Jian-Jun Li

**Affiliations:** ^1^ Department of Radiotherapy, Hainan General Hospital, Hainan, China; ^2^ Research and Education Department, Hainan General Hospital, Hainan, China; ^3^ Department of Radiology, Hainan General Hospital, Hainan, China; ^4^ Siemens Healthcare, MR Scientific Marketing NE Asia, Beijing, China

**Keywords:** diffusion-kurtosis imaging, DKI, magnetic romance imaging, MRI, nasopharyngeal carcinoma, NPC, radiotherapy

## Abstract

In this prospective study, we analyzed diffusion kurtosis imaging (DKI) parameters to predict the early response to radiotherapy in 23 nasopharyngeal carcinoma (NPC) patients. All patients underwent conventional magnetic resonance imaging (MRI) and DKI before and after radiotherapy. The patients were divided into response (RG; no residual tumors; 16/23 patients) and no-response (NRG; residual tumors; 7/23 patients) groups, based on MRI and biopsy results 3 months after radiotherapy. The maximum diameter of tumors in RG and NRG patients were similar prior to radiotherapy (*p*=0.103). The pretreatment diffusion coefficient (D) parameters (D_axis_, D_mean_ and D_rad_) were higher in RG than NRG patients (*p*=0.022, *p*=0.027 and *p*=0.027). Conversely, the pre-treatment fractional anisotropy (FA) and kurtosis coefficient (K) parameters (K_axis_, K_fa_, K_mean_, K_rad_ and Mkt) were lower in RG than NRG patients (*p*=0.015, *p*=0.022, *p*=0.008, *p*=0.004, *p*=0.001, *p*=0.002). The K_rad_ coefficient (0.76) was the best parameter to predict the radiotherapy response. Based on receiver operating characteristic curve analysis K_rad_ showed 71.4% sensitivity and 93.7% specificity (AUC: 0.897, 95% CI, 0.756-1). Multivariate analysis indicated DKI parameters were independent prognostic factors for the short-term effect in NPC. Thus, DKI predicts the early response to radiotherapy in NPC patients.

## INTRODUCTION

Radiotherapy is the most frequent treatment for patients with nasopharyngeal carcinoma (NPC), which is one of the most common malignant tumors in Southeast Asia [1]. However, regional residue or recurrence because of radioresistance results in treatment failure in many cases [2, 3]. The 3-year and 5-year local control, disease-free survival, and overall survival rates were 83.3%, 82%, 83.8%, and 76.1%, 73.2%, 76.3% respectively [4]. Therefore, early prediction of radiotherapy response is of paramount importance. It would help clinicians to shift to personalized medicine and avoid unnecessary systemic toxicity for NPC patients. Prospective studies have used diffusion-weighted imaging (DWI) pre-treatment to analyze treatment response in head and neck tumors [5–9]. The mono-exponential DWI analyzes *in vivo* water diffusion based on standard Gaussian distribution [10]. However, *in vivo* water diffusion is complicated because barriers like cell membranes and tumor heterogeneity. In contrast, diffusion kurtosis imaging (DKI) is based on non-Gaussian diffusion model [11]. It can specifically measure tissue structure including cellular compartments and membranes [12]. DKI performs better in assessing central nervous system diseases such as cerebral glioma [13], Parkinson’s disease [14], and idiopathic generalized epilepsy [15] compared to DWI. Additionally, DKI is preferred for investigating abnormalities in tissues with isotropic structure such as gray matter, where techniques like diffusion tensor imaging (DTI) are less applicable [16]. DKI also clarifies the tumor microstructural details, thereby providing useful information to analyze the treatment effects [16].

NPCs demonstrate high cellular heterogeneity at the molecular level [17]. A single NPC tumor has multiple cellular components, including tumor xenografts derived from homogeneous cell populations with the same genetic background [17]. Chen *et al.* demonstrated that DKI enabled predicting the effect of neoadjuvant chemotherapy in NPC patients [18]. Based on these aspects, we hypothesized that DKI would be superior to DWI and DTI in assessing treatment response in NPC. Therefore, in this study, we explored if DKI would enable early prediction of the radiotherapy response in NPC patients.

## RESULTS

### Radiotherapy treatment outcome

Three months after the end of radiotherapy, 16 (69.6%) response group (RG) patients had no residual tumors, whereas 7 (30.4%) no-response group (NRG) patients had residual tumors. The intra-class correlation coefficient of DKI parameters was 0.78.

### Analysis of radiotherapy response prediction by DKI

The maximum diameter of pre-treatment tumors in NRG patients was larger than RG patients, but not statistically different (p=0.103). Also, age and T stage distributions were similar between patients in the two groups (p=0.079, p=0.222).

**Figure 1:**
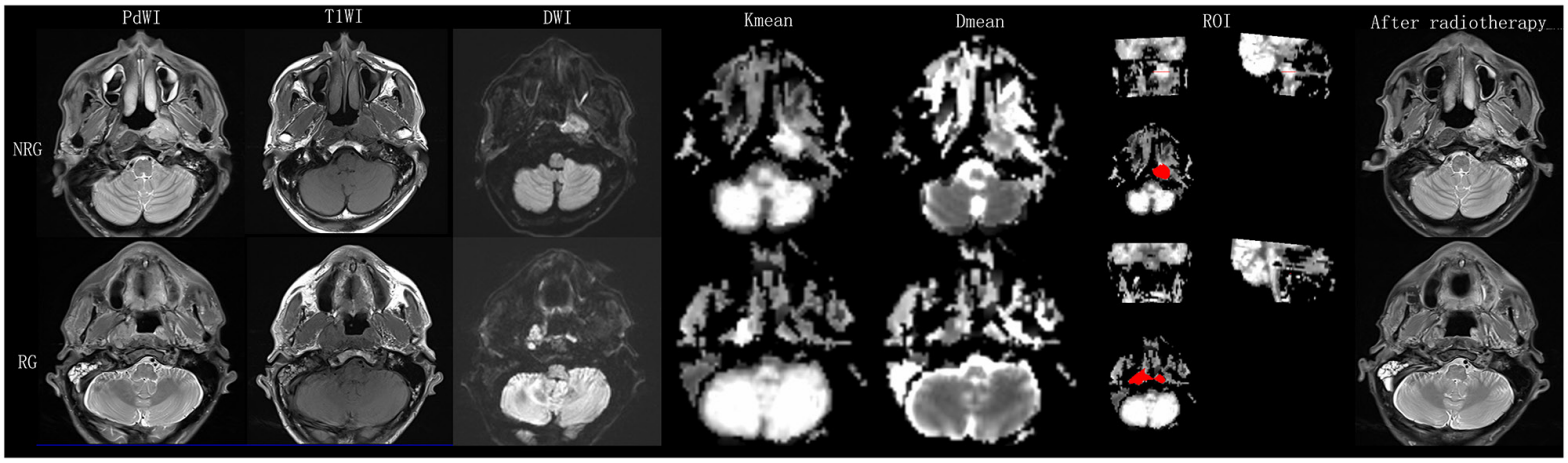
The first row shows a 62-year-old non-responder NPC patient The axis T1, PD and DWI show the lesions located at the left nasopharyngeal wall and cavum. The manually drawn ROI within the boundaries of the NPC on K_mean_ map is also shown. The maximum diameter of the tumor was 3.5 mm^2^ before radiotherapy. Residual tumor was detected after radiotherapy. D_mean_ and K_mean_ values were 1.48 x 10^-3^ mm^2^/s and 0.72 before treatment. The second row shows a 63–year-old responder NPC patient. The axis T1, PD and DWI show the lesion affecting the bilateral mucous membrane of the nasopharynx. The maximum diameter of the tumor was 3.09mm^2^ before radiotherapy. No residual tumor was detected after radiotherapy. D_mean_ and K_mean_ values were 1.22 x 10^-3^ mm^2^/s and 0.83 before treatment. Note: NRG: non response group; RG: reponse group.

Among the nine DKI parameters, pre-treatment D parameters (D_axis_, D_mean_ and D_rad_) were larger in RG compared to NRG patients (*p*=0.022, *p*=0.027 and *p*=0.027). The pre-treatment FA, and K parameters (K_ax_, K_fa_, K_mean_, K_rad_ and Mkt) of the RG group were lower compared to NRG (*p*=0.015, *p*=0.022, *p*=0.008, *p*=0.004, *p*=0.001, *p*=0.002; Table 1 and Figure 1).

**Table 1:** Patient characteristics

Characteristic	No. of patients		Total	%	No. of patients		Total	%
	Stage I-II	Stage III-IV			RG	NRG		
**Sex**								
**M**	5	12	17	73.9%	12	5	17	73.9%
**F**	2	4	6	26.1%	4	2	6	26.1%
**Age**								
**<45**	0	6	6	26.1%	4	4	8	34.8%
**≥45**	7	10	17	73.9%	12	3	15	65.2%
**Pathologhical type**								
**Undifferentiated**	3	17	20	87%	14	6	20	87%
**Differentiated**^**a**^	2	1	3	13%	2	1	3	13%
**AJCC T stage**								
**T1**	0	0	0	0%	0	0	0	0%
**T2**	7	5	12	52.2%	7	1	8	34.8%
**T3**	0	8	8	34.8%	8	3	11	47.8%
**T4**	0	3	3	13%	1	3	4	17.4%
**AJCC N stage**								
**N0**	1	0	1	4.3%	1	0	1	4.3%
**N1**	6	0	6	26.1%	6	0	6	26.2%
**N2**	0	11	11	47.8%	5	6	11	47.8%
**N3**	0	5	5	21.7%	4	1	5	21.7%

a: WHO classification. M: male, F: female; AJCC: American Joint Committee on Cancer.

RG: response group; NRG: non-response group.

**Figure 2:**
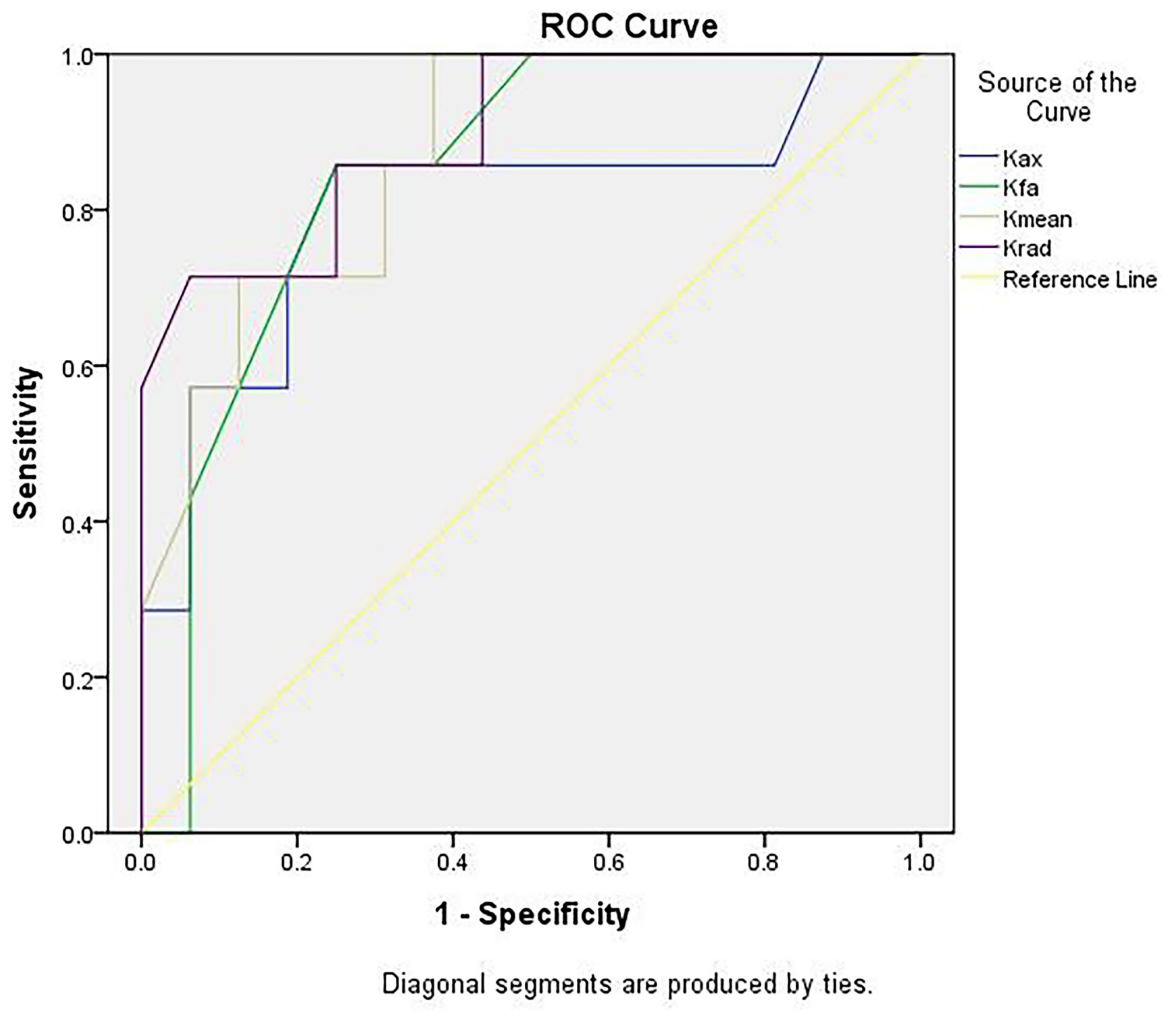
ROC curve analysis of DKI specificity and sensitivity in radiotherapy AUC = 0.871 for K_FA_; AUC = 0.871 for K_mean_; AUC = 0.897 for K_rad_; and AUC:0.893 for MTK.

Kurtosis parameters (K_fa_, K_mean_ K_rad_ and Mkt) were chosen for Receiver operating characteristic (ROC) analysis. Table 2 shows the comparison of Kurtosis parameters performed to predict radiotherapy response in NPCs. K_rad_ performed better than other parameters in accurately predicting the radiotherapy response (Figure 2).

**Table 2:** Parameters between responder group (RG) and nonresponder group (NRG)

Parameter	RG	NRG	*P* value
**Diameter (cm)**	2.97±1.13	3.31±0.57	0.103
D_ax_ (*10^-3^m^2^/s)	2.01±0.51	1.53±0.32	0.022
D_mean_(*10^-3^m^2^/s)	1.75±0.48	1.31±0.26	0.027
D_rad_(*10^-3^m^2^/s)	1.64±0.48	1.20±0.24	0.027
**FA**	0.15±0.03	0.17±0.02	0.015
**K_ax_**	0.55±0.18	0.79±0.24	0.022
**K_fa_**	0.24±0.08	0.32±0.04	0.008
**K_mean_**	0.55±0.16	0.79±0.12	0.004
**K_rad_**	0.53±0.14	0.76±0.1	0.001
**Mkt**	0.57±0.15	0.83±0.12	0.002

### Multivariate analysis of factors affecting short-term radiotherapy response

Binary logistic regression analysis indicated that age, clinical stage and DKI parameters were independent prognostic factors for the short-term effect in NPC when the presence of a residual local tumor was assessed as a dependent variable. The advanced T stage (T3-4) was associated with increased risk of a local residual tumor, whereas lower K values (K_fa_, K_mean_, K_rad_ and Mkt) were associated with reduced risk of a local residual tumor.

## DISCUSSION

The apparent diffusion coefficient (ADC) for predicting local outcomes for NPC patients have been described previously [5, 23, 24]. Also, the DTI or DKI parameters for predicting treatment outcomes in head and neck lesions have been described in previous studies [12]. However, DKI parameters have not been used to assess radiotherapy response in NPC patients. Our study show that tumors that respond better to radiotherapy have higher D parameter values (D_mean_, D_rad_, D_axis_) or low values for FA, K_axis_, K_mean_ and K_rad_. These results demonstrate the feasibility of DKI in predicting radiotherapy response in NPC patients.

It is controversial if ADC values derived from DWI predict treatment outcomes in NPC patients accurately [25, 26]. Some studies found a clear correlation between baseline ADC values and treatment response in NPCs [8, 26], whereas other studies showed more moderate or insignificant results [23]. Zhang *et al.* showed that pre-treatment ADC was an independent prognostic factor for local control and disease-free survival [8]. ADC showed 65.2% sensitivity and 69.5% specificity to distinguish local failure, which was lower than the DKI parameters reported in our study. However, Chen *et al.* reported no significant differences in pre-treatment ADC between stage III-IV NPC responders and non-responders after neoadjuvant chemotherapy [23].

Different diffusion models have been devised to analyze the complicated non-Gaussian diffusion behaviour of biological tissues and acquire information regarding various tissue properties [27, 28]. DKI model is an alternate method that can provides tissue heterogeneity and diffusion data simultaneously. It was found to be more reliable and reproducible than mono-exponential and bi-exponential models [27]. Jing Yuan *et al* used the same b-value (0, 500, 1000, 1500 sec/mm^2^) used in this study and reported that non-Gaussian diffusion models including DKI performed better than mono-exponential model in comparing the NPC lesions to surrounding tissues [29].

DKI also provides more information regarding microstructure compared to other non-Gaussian diffusion models such as DTI. Jensen *et al.* studied on phantom, which was essentially isotropic and demonstrated that a non-zero diffusional kurtosis did not require diffusional anisotropy [16]. The non-zero diffusional kurtosis observed in tumor or gray matter had similar origin. However, the association between DKI parameters and the diagnosis of local control is complex and unclear [12, 20]. We postulate that K and D values reflect tissue microstructural complexity (tumor cell density, stromal volume of the tumor tissue, and the complexity of the membrane structure) when analyzed in detail by a multiple b-value with a nonlinear fitting model. Therefore, DKI parameters reflect the damage to tumor tissue at microstructural level, thereby enabling early prediction of the treatment response.

Binary logistic regression analysis indicated that age, clinical stage and DKI parameters were independent factors for short-term effect in NPC. However, two-tailed Fischer’s exact t-test showed that age and T-staging were similar between the two groups. One reason for this conflict could be that parameters such as old age and T-stage affect the general health conditions of the patients, thereby affecting radiotherapy response and tolerance. However age and T-staging did not reflect the biological behavior of tumors. DKI analysis reflected the microstructure of tumor cells and treatment response. Hence, DKI parameters predict treatment response better than clinical indicators. Among the DKI parameters, K parameters were better at predicting radiotherapy response than the D parameters. These findings were consistent with previous reports [22, 30]. K parameters represent the excess diffusion kurtosis in the tissue and probably reflect the microstructural complexity of tissues [16]. Therefore, K parameters may potentially be more sensitive to pathological changes. Although macroscopic necrosis and cystic lesions were rare in NPC, micro-necrotic areas and tissue heterogeneity varied in different NPC tumors to different degrees [31]. Lesions with more heterogeneity due to hypoxia showed poorer sensitivity to radiotherapy. Therefore, the K parameters predicted the radiotherapy response better than D parameters.

K parameters have not been used in prediction studies previously. Quentin *et al* showed that K_mean_ and K_axis_ distinguished prostate cancer from prostatitis, the peripheral zone or the central gland compared to K_rad_ [22]. However, in our study, the values for all the K parameters were different between the two groups (RG and NRG) with K_rad_ being the most sensitive K parameter to differentiate radiotherapy response. The possible explanation could be that NPC tended to grow along the nose pharynx mucosa. The lesion spread more axially and actively, which meant that the axial architecture changed significantly due to the tumor. When NPC spread to surrounding tissues, the higher T stage showed significant heterogeneity. FA and K_FA_ were also significantly different between RG and NRG. Li *et al.* showed that FA and ADC values could detect invasion of the trigeminal nerve at early stage in NPC patients [32]. Mkt and K_FA_ values reflect the anisotropy of the kurtosis tensor without contributions from the diffusion tensor [33, 34]. These parameters provide additional information regarding deep brain structures and are particularly advantageous for assessing diffusion in complex tissue environments [35]. In this study, Mkt and K_FA_ showed differences between the two patient groups. However, further studies are required to confirm the significance of these parameters in head and neck tumors.

Our results showed lower D values in the NRG than RG, which was opposite to some previous results of ADC for head and neck cancers [36]. However, our results were consistent with one ADC study [37] and a new study of DKI in nasal or sinonasal squamous cell carcinoma patients [12]. The possible reasons are (1) D values were influenced by K values and are negatively correlated. (2) The diffusion coefficient D was likely to be more sensitive to reflect heterogeneity than ADC probably due to necrosis proliferation and hypoxia [38]. However, our current study design did not incorporate hypoxia measurements. (3) Diffusion coefficient maybe related to T-stage. As T-stage increases, diffusion coefficient values gradually decrease. High T stage and lower diffusion coefficient values tended to show low radiosensitivity [37]. Future studies with pathology might shed more light on these aspects.

This study has two main limitations. (1) Radiotherapy response was determined at 3 months after radiotherapy. Therefore, DKI parameters for long term radiotherapy response such as mortality and recurrence rates were not elucidated. We chose 3 months as the local control time point in NPCs similar to the study by Hong *et al.* [21]. In order to avoid residual lesions that are common at this early stage, we used biopsy to confirm MRI results. However, it would be difficult to justify a change in therapy at the outset of treatment based on this endpoint. Therefore, a longer follow-up period is necessary to determine if the DKI parameters correlate with progression-free survival and long term curative effects. (2) This was a single center study with small sample size. Therefore, multi-center trials with larger sample sizes are needed to confirm our findings.

## MATERIALS AND METHODS

### Study subjects and eligibility criteria

This study was approved by the Hainan General Hospital Ethics Committee. All patients provided written informed consent. The criteria for eligibility for patients to be included in this study were: (1) at least 18 years of age; (2) pathological diagnosis of NPC; (3) Karnofsky score >80, and (4) no treatment prior to the MRI examination for NPC. The exclusion criteria were as follows: (1) contraindication for MRI; (2) diagnosis of other malignant tumors in the past 5 years; (3) failed systematic radiotherapy; (4) distant metastasis; (5) radiotherapy to the head and neck region in the past.

Twenty-six consecutive NPC patients treated at the Hainan General Hospital from November 2014 through August 2016 were recruited in this prospective study. Two patients never started radiotherapy, and one withdrew from the study due to personal reasons. Thus, the final group comprised 23 participants. Table 3 shows the patient characteristics and stage of disease. The TNM status was determined according to the latest 7th edition of the American Joint Committee on Cancer (AJCC) staging system [19]. Staging was performed by MRI and CT-scan of the head and neck, ultrasound of the abdomen, CT-scan of the thorax and a bone scan. Advanced stage was predominantly seen at diagnosis.

**Table 3:** Compare of kurtosis paremeters to predict radioterapy response in NPCs

Parameter	Cut value	Sensitivity	Specificity	AUC	(95% CI)
**K_fa_**	0.3	85.7%	75%	0.871	0.722-1
**K_mean_**	0.62	100%	62.5%	0.871	0.722-1
**K_rad_**	0.76	71.4%	93.7%	0.897	0.756-1
**Mkt**	0.81	71.4	93.7%	0.893	0.756-1

AUC: area under the ROC Curve; CI: confidence interval.

### Radiotherapy treatment and response evaluation

All NPC patients underwent radiotherapy with curative intent. The protocol for nasopharynx and neck radiotherapy involved 3D-conformal intensity-modulated radiation therapy. The total dose of nasopharynx radiotherapy was 68.2-72.6 Gy, which was divided into 31 to 33 fractions in 43 to 54 days.

The short-term curative effect of radiotherapy was examined three months after the completion of radiotherapy. Patients with no residual tumor or no local bulges due to thickening of the mucous membrane of the nasopharynx on MRI were not subjected to biopsy. If a residual tumor was detected on MRI, however, electronic nasopharyngoscopy (biopsy) under the guidance of imaging were performed to confirm the results. Patients with residual tumor were then classified into NRG and RG.

### MR scan protocol

All patients were imaged by a 3T clinical MR imaging scanner (MAGNETOM Skyra, Siemens Healthcare, Erlangen, Germany) placed in the supine position with a 8-channel head and neck array coil during pre-treatment and 3^rd^ month after radiotherapy. Patients were instructed to avoid motion, swallowing and talking. MRI examinations included proton density-weighted imaging (PDWI) and DKI sequences. Axial turbo spin echo (TSE) PDWI was obtained using the short time inversion recovery (STIR) technique. A fat-suppressed single-shot spin echo planer (SE-EPI) sequence was used in the axial plane using 30 orthogonal diffusion directions for DKI examination. DKI parameters, including fractional anisotropy (FA), mean diffusion coefficient (D_mean_), mean kurtosis coefficient (K_mean_), mean kurtosis tensor (Mkt), kurtosis fractional anisotropy (K_fa_), both the diffusion coefficient and the kurtosis in radius and axis (D_rad_, D_axis_, K_rad_, K_axis_), were measured pretreatment using software based on the diffusion signals fitted by least square methods nonlinearly: S_b_/S_0_=exp[-b*ADC_0_+K*(bADC_0_)^2^/6] [20, 21]. The scan parameters for PDWI were: echo time (TE), 20msec; repetition time (TR), 5200msec; field of view (FOV), 320*320mm; slice thickness/gap, 4/1mm; number of slices, 25; number of signal averages (NSA), 2; scan time, 2:04min. The scan parameters for axial DKI examination were: TE, 72msec; TR, 8300msec; FOV, 230*240mm; slice thickness/gap, 4/1mm; number of slices, 25; voxel size, 2.2*1.5mm; reconstruction matrix, 224; IR delay, 240msec; NSA, 2; iPAT factor, 3; water-fat shift, minimum; recon voxel size, 1.24mm; b-values, 0, 500, 1000, 1500 sec/mm^2^; and total duration time of MR examination, 10:57min.

### DKI data processing and measurements

To characterize the non-Gaussian diffusion behavior in complex tissues, the signal intensity versus b values from each voxel in the ROI were extracted to fit the non-Gaussian model as S_b_/S_0_=exp [-b*ADC_0_+K*(bADC_0_)^2^/6] [20, 21].

DKI parameters, including fractional anisotropy (FA), mean diffusion coefficient (D_mean_), mean kurtosis coefficient (K_mean_), mean kurtosis tensor (Mkt), kurtosis fractional anisotropy (KFA), both the diffusion coefficient and the kurtosis in radius and axis (D_rad_, D_axis_, K_rad_, K_axis_), were measured with the Diffusional Kurtosis Estimator software tool (DKE, Version 2.6, built on Feb 25, 2015) [22]. DKI parametric maps including fractional anisotropy (FA), corrected diffusion coefficients (D parameters including D_axis_, D_mean_, D_rad_), and principle kurtosis eigenvectors (K parameters including K_axis_, K_fa_, K_mean_, K_rad_ and Mkt) were calculated with DKE software. Regions of interest (ROI)-based measurements were performed in the parameter maps using the MRIcro software (www.mricro.com, Version 1.40, Chris Rorden, University of South Carolina, USA). The images were reviewed by a MRI physicist (Y-M Z) with 20 years experience and a radiologist (W-Y H) with 9 years experience in head and neck MRI, respectively. Both were blinded to the clinical history of the patients and their response to radiotherapy. ROIs were manually drawn on the maximum cross-sectional area of the primary lesions using axial Pd-weighted images as reference. ROIs were copied in different parametric maps, which were derived from the K_mean_ map to ensure that the same areas were evaluated always. The maximum diameter of a primary lesion was measured in axial PDWI. All parameter values were the mean values obtained by two radiologists independently.

### Statistical analysis

SPSS for MAC (Version 22; IBM SPSS) was used for data analysis. The intra-class correlation coefficient was calculated for parameters measured by the two radiologists. Continuous quantitative variables were compared with Mann-Whitney U-test. Two-tailed Fischer’s exact test was used to compare age (age<45 versus age≥45) and T stage (T1-2 versus T3-4) parameters between two groups. ROC curve analyses were performed to characterize the predictive value of each parameter regarding radiotherapy response in NPC patients. Predictive performance was determined by calculating the area under the ROC curve (AUC). The sensitivity and specificity of the predictions were calculated. Cutoff values were established by calculating the maximal Youden index (Youden index=sensitivity+specificity-1). Binary logistic regression was performed to determine factors that predicted radiotherapy response independently. A *p*<0.05 was considered statistically significant.

## CONCLUSION

In conclusion, we demonstrate that DKI is a noninvasive tool to predict the early response to radiotherapy in NPC patients.

## Abbreviations

ADC: apparent diffusion coefficient
AJCC: American Joint Committee on Cancer
AUC: area under the curve
DKE: diffusional kurtosis estimator
DKI: diffusion kurtosis imaging
Dmean: mean diffusion coefficient
DTI: diffusion tensor imaging
DWI: diffusion-weighted imaging
EPI: echo planer imaging
FA: fractional anisotropy
FOV: field of view
MRI: magnetic romance imaging
KFA: kurtosis fractional anisotropy
Kmean: mean kurtosis coefficient
Mkt: mean kurtosis tensor
NPC: nasopharyngeal carcinoma
NRG: no-response group
NSA: number of signal averages
PDWI: proton density-weighted imaging
RG: response group
ROC: receiver operating characteristic
SE: spin echo
STIR: short time inversion recovery
TE: time of echo
TR: time of repetition
TSE: turbo spin echo
